# Supplementary material for: Antimicrobial proteins from oyster hemolymph improve the efficacy of conventional antibiotics
Source: PLoS One. 2025 Jan 21;20(1):e0312305. doi: 10.1371/journal.pone.0312305 (PMC11750097; doi:10.1371/journal.pone.0312305)
Supplement: S1 File — (DOCX) [file pone.0312305.s001.docx]

**Antimicrobial proteins from oyster hemolymph improve the efficacy of conventional antibiotics**

Kate Summer, Lei Liu, Qi Guo, Sarah Giles, Bronwyn Barkla, and Kirsten Benkendorff

**SUPPLEMENTARY TABLES**

**Table S1:** Bacterial species and strains, growth media and controls used in the study.

| **Species** | **Strain** | **Characteristics and resistance; rationale for selection** | **Agar** | **Media** | **Inoculum colonies (*n*) and incubation time (h)** | **Positive control antibiotic** | **Positive control MIC/MBC µg/mL (CLSI)** |
| --- | --- | --- | --- | --- | --- | --- | --- |
| **Gram positive** | |  |  |  |  |  |  |
| *Streptococcus pneumoniae* | ATCC 51916 | Multidrug-resistant including to extended-spectrum cephalosporins, widely used for antimicrobial susceptibility testing against drug-resistant pathogens | HBA | CAMHB + 5% LHB | *n*=2, 3.5 h | Ampicillin | <0.25 |
| *S. pneumoniae* | Clinical isolates 6B, 14, and 19F | Associated with resistance and clinical disease, covered by PCV13 | HBA | CAMHB + 5% LHB | *n*=2, 3.5 h | Ampicillin | <0.25 |
| *Streptococcus pyogenes* | ATCC 19615 | Used widely as a control strain for Strep A as two emm (M protein) genes are encoded, serotypes 5 and 49, which have been associated with throat and skin infections, respectively | HBA | CAMHB + 5% LHB | *n*=2, 3.5 h | Ampicillin | <0.25 |
| *Staphylococcus aureus* | ATCC 25923 | Quality control strain for *S. aureus* susceptibility testing; more potential to detect novel antimicrobial activity than MRSA strains | Nutrient | CAMHB | *n*=1, 3.5 h | Ampicillin | <2.0 |
| **Gram negative** | |  |  |  |  |  |  |
| Non-typeable *haemophilus influenzae* (NtHi) | ATCC 10211 | Quality control strain for Nt*Hi* susceptibility testing | Choc | BHI + hemin (10 ug/mL) + β-NAD, 10 ug/mL) | *n*=2, 1.5 h | Ampicillin | 1.0-4.0 |
| *Moraxella catarrhalis* | Clinical isolate K65 | Non-clumping variant; clinically relevant | Choc | BHI + hemin (10 ug/mL) + β-NAD, 10 ug/mL) | *n*=3, 5 h | Ciprofloxacin | <1.0 |
| *Klebsiella pneumoniae* | ATCC 13883 | Recommended for susceptibility and resistance testing | Nutrient | CAMHB | *n*=1, 1 h | Trimethoprim | 8.0-16.0 |
| *Pseudomonas aeruginosa* | Clinical isolate 385 | Mucoid phenotype characteristic of isolates from cystic fibrosis patients; clinically relevant | Nutrient | CAMHB | *n*=1, 3.5 h | Gentamycin |  |

ATCC: American Type Culture Collection strain

BHI: Brain-heart infusion (BD Bacto™ powder, Thermo Fisher) prepared according to manufacturer’s instructions

CAMHB: Cation-adjusted Mueller Hinton II broth (BD BBL™ powder, Thermo Fisher) prepared according to manufacturer’s instructions

Choc: chocolate agar

CLSI: Clinical Laboratory Standards Institute

HBA: horse blood agar

LHB: lysed horse blood; defibrinated horse blood (Edwards Group, Australia) lysed over five freeze-thaw cycles, added at 5% v/v to autoclaved/cooled CAMHB; final concentration in assays 2.5%

MIC: minimum inhibitory concentration

β-NAD: beta nicotinamide adenosine dinucleotide

PCV13: pneumococcal conjugate vaccine

**Table S2:** Effective biofilm inhibitory concentrations (µg/mL) of HPE and conventional antibiotics against a range of pathogens presented as MBC: minimum inhibitory concentration (100% relative to the growth control) (± standard deviations) and EC_50_: median effective concentration (with 95% highest posterior density intervals [HPDI]). Effective concentrations are for antibiotics in the presence of HPE at sub-MIC concentrations (1-3 µg/mL) for susceptible species (*Streptococcus*) and at higher concentrations (6-12 µg/mL) for non/less-susceptible species (*S. aureus*, and Gram-negative sp.) NT: not tested, NC: not calculable.

|  |  | | **Effective biofilm inhibitory concentrations (µg/mL)**  **Minimum 100% biofilm inhibitory concentration** (±SD),  EC_50_ (95% HPDI’s) | | | | |
| --- | --- | --- | --- | --- | --- | --- | --- |
| **Species (strain)** | | **Antibiotic** | | **HPE individual treatment** | **Antibiotic individual treatment** | **Antibiotic**  **+ 1 µg/mL HPE combination treatment** | **Antibiotic**  **+ 3 µg/mL HPE combination treatment** |
| **Gram-positive** | | | |  |  |  |  |
| *S. pneumoniae* (ATCC 51916) | | Ampicillin | | **4.82** (±0.0),  2.96 (2.45, 3.91) | **0.23** (±0.09),  0.13 (0.10, 0.18) | **0.125** (±0.07),  0.063 (0.032, 0.12) | **0.063** (±0.0),  0.04 (0.03, 0.06) |
| *S. pneumoniae* (19F, clinical) | | Ampicillin | | **4.82** (±0.0),  1.23 (0.07, 13.85) | **0.23** (±0.03),  0.03 (0.01, 0.12) | **0.02** (±0.0),  0.003 (0.001, 0.008) | **0.01** (±0.0),  0.001 (0.001, 0.002) |
| *S. pneumoniae* (14, clinical) | | Ampicillin | | **2.41** (±0.0),  0.10 (0.02, 0.48) | **0.13** (±0.0),  0.06 (0.06, 0.08) | **0.02** (±0.0),  0.003 (0.001, 0.008) | **0.004** (±0.0),  0.002 (0.002, 0.002) |
| *S. pneumoniae* (6B, clinical) | | Ampicillin | | **9.63** (±0.0),  2.50 (0.70, 5.70) | **0.10** (±0.03),  0.04 (0.03, 0.06) | **0.016** (±0.0),  0.009 (0.008, 0.011) | **0.08** (±0.0),  0.005 (0.004, 0.006) |
| *S. pyogenes* (ATCC 19615) | | Ampicillin | | **19.27** (±11.80),  3.59 (2.62, 4.69) | **0.13** (±0.0),  0.03 (0.01, 0.06) | NT | **0.05** (±0.01),  0.004 (0.002, 0.009) |
|  | |  | |  |  | **Antibiotic**  **+ 6 µg/mL HPE combination treatment** | **Antibiotic**  **+ 12 µg/mL HPE combination treatment** |
| *S. aureus* (ATCC 25923) | | Ampicillin | | NC,  19.07 (9.07, 39.10) | 0.58 (±0.03) | NT | 0.09 (±0.04) |
| **Gram-negative** | | | |  |  |  |  |
| Nt*Hi* (ATCC 10211) | | Ampicillin | | NC | NC | NT | NC |
| *M. catarrhalis* (ATCC K65) | | Ciprofloxacin | | NC | NC | NT | NC |
| *K. pneumoniae* (ATCC 51916) | | Trimethoprim | | NC | NC | NT | NC |
| *P. aeruginosa* (385, clinical) | | Gentamicin | | NC,  2.53 (1.50, 5.02) | 3.0 (±1.0),  1.33 (1.07, 1.74) | 1.50 (±0.50),  0.37 (0.19, 0.75) | 1.0 (±0.0),  0.29 (0.14, 0.62) |

**Table S3:** The most effective molluscan extracts and compounds (MECs) tested for *in vitro* antibacterial activity (against at least one of the following: *S. pneumoniae*, *S. pyogenes*, *S. aureus*, *P. aeruginosa*, *M. catarrhalis*, Nt*Hi*), based on our earlier work [1, 2] and now including the results from this study (shown in red). Excludes preliminary data (from disc diffusion assays) and MECs with effective concentrations ≥300 µg/mL. EC_50_: 50% effective concentration; MIC: minimum inhibitory concentration; MBC: minimum bactericidal concentration; AMP: antimicrobial peptide; G+/G-: Gram positive/negative.

| Mollusc class  Family | Derivative part | Specific MEC | Effective concentrations | References |
| --- | --- | --- | --- | --- |
| Bivalvia |  |  |  |  |
| Margaritidae | Hemolymph | Structurally altered histone-derived AMP | Significant reduction in growth at 200 ug/mL | [3] |
| Mytilidae | Sperm | Crude perchloric acid extract and 3 isolated protamine-like proteins | MICs 7.8–250 μg/mL, MBCs (μg/mL) 15.7–250 μg/mL | [4] |
|  | Hemolymph | Myticin C and 9 peptide fragments | MICs 32 μM for 3 peptide fragments | [5] |
|  | Hemolymph | Myticin A and B peptides | MBCs 1 to >20 μM | [6] |
| Ostreidae | Hemolymph | HPE | MBCs: 4.4-24.1 µg/mL; 1-12 µg/mL in combinations | This study |
|  | Gills | Defensin | MICs 2.4 to 15 µg/mL | [7] |
|  | Hemolymph | URP20 (upregulated peptide) | MICs 1 to 10 μM and MBCs 5 to 20 μM | [8] |
|  | Gills | CgMolluscidin | MICs 0.4 to 31.3 μg/mL | [9] |
|  | NA | Recombinant defensin MgDefdg | 50 µg/mL had 70 to 99% antibacterial activity | [10] |
|  | Body | CgPep33 obtained by enzyme digestion | EC50s 18.6 to 48.2 μg/mL | [11] |
|  | NA | BigDef1 (Cg-BigDef1) and separate domains. | MICs 0.15 to >10 mM | [12] |
|  | NA | Recombinant proline-rich peptide (CgPrp) and defensin (CgDef) | MICs 5 to 75 µg/mL | [13] |
|  | NA | Synthesised CgPrp and Cg-Def | MICs 0.005 to >20 μM (G+) but no/low activity against G- | [14, 15] |
| Gastropoda |  |  |  |  |
| Achatinidae | Mucus | Mytimycin-AF (antimicrobial peptide) | MICs 1.9 to 30 μg/mL | [16] |
| Fissurellidae | Body | Scutinin A and B | MICs 30-100 μg/mL | [17] |
| Helicidae, Muricidae | Hemolymph | Experimentally purified hemocyanins and subunits (βc-HaH and RvH1 | MICs 6.5 μM βc-HaH; 1.25–10 μM RvH1; | [18] |
|  | Hemolymph | Hemolymph peptides | Up to 80% growth inhibition with 113–598 μg/mL | [19] |
|  | Body | Acid-acetone peptide extract | MIC: 39.06 ug/mL (bacteriostatic); 50% reduction in biofilm formation at 39 ug/mL, 2.5 mg/mL to degrade pre-formed biofilm | [20] |
| Onchidiidae | Body | Dolabellanin B2 (AMP) | MICs 10–25 ug/mL | [21] |
| Truncatellidae | Body (symbiotic *Streptomyces* sp.) | 7,8-dideoxygriseorhodin C (DC) | MICs 0.08–0.12 μg/mL; combination treatments improved efficacy of oxacillin | [22] |

**Table S4:** Identified proteins with high abundance in HPE/Fraction 7 (relative to fractions HF6 and HF8) in this study and previous work. Candidates for the proteins responsible for antimicrobial activity show high abundance in HPE collected in both studies, were less abundant with 60°C heat treatment (causing a loss in activity), and have strong available evidence for antimicrobial activity (i.e., complete green rows). Strength of available evidence for antimicrobial activity is based on data provided in this study and Summer et al., (2004).

| **Identified protein** | **Abundant in HPE (Fraction 7) in Summer et al., 2004** | **Abundant in HPE in this study** | **Change in abundance with heat treatment** | **Literature relevant to antimicrobial activity** | **Overall strength of evidence** |
| --- | --- | --- | --- | --- | --- |
| - 40S ribosomal protein S15 |  | Yes |  |  |  |
| - 40S ribosomal protein S25 |  | Yes |  |  |  |
| - 40S ribosomal protein S28 (Fragment) |  | Yes |  |  |  |
| - 40S ribosomal protein S4 |  | Yes |  |  |  |
| - 60S ribosomal protein L11 |  | Yes |  |  |  |
| - 60S ribosomal protein L12 |  | Yes |  |  |  |
| - 60S ribosomal protein L14 |  | Yes |  |  |  |
| - 60S ribosomal protein L23a-like |  | Yes |  |  |  |
| - 60S ribosomal protein L24-like |  | Yes |  |  |  |
| - 60S ribosomal protein L6 |  | Yes |  |  |  |
| - ALMS_motif domain-containing protein | Yes |  |  |  |  |
| - Carbonic anhydrase 1-like | Yes | Yes | Lower | Some | Intermediate |
| - Catchin protein |  | Yes |  |  |  |
| - CD109 antigen-like isoform X3 |  | Yes |  |  |  |
| - Cofilin | Yes |  |  |  |  |
| - Creatine kinase |  | Yes |  |  |  |
| - Cystatin B-like protein | Yes | Yes | Lower | Yes | High |
| - Cystatin-A1-like |  | Yes |  |  |  |
| - Extracellular superoxide dismutase [Cu-Zn]-like |  | Yes |  |  |  |
| - Extracellular superoxide dismutase | Yes | Yes | Higher | Some | Low |
| - Fructose-bisphosphate aldolase | Yes |  |  |  |  |
| - Gelsolin-like protein 2 isoform X3 | Yes |  |  |  |  |
| - Gelsolin-like protein 2 | Yes |  |  |  |  |
| - Heterogeneous nuclear ribonucleoprotein A/B | Yes |  |  |  |  |
| - Taurocyamine kinase-like (low quality) |  | Yes |  |  |  |
| - Muscle LIM protein 1-like |  | Yes |  |  |  |
| - Myosin heavy chain, striated muscle | Yes |  |  |  |  |
| - Myosin heavy chain, striated muscle-like isoform X1 | Yes |  |  |  |  |
| - Myosin heavy chain, striated muscle-like isoform X2 | Yes | Yes |  |  |  |
| - Nascent polypeptide-associated complex subunit α | Yes |  |  |  |  |
| - PDZ and LIM domain protein Zasp-like | Yes | Yes | Lower | No | Low |
| - Peptidyl-prolyl cis-trans isomerase | Yes | Yes | Lower | No | Low |
| - Peroxiredoxin 6 |  | Yes |  |  |  |
| - Putative 40S ribosomal protein S14 |  | Yes |  |  |  |
| - Severin | Yes | Yes | No change | No | Low |
| - SH3 domain-binding glutamic acid-rich protein | Yes |  |  |  |  |
| - SOCS box domain-containing protein | Yes |  |  |  |  |
| - Transgelin-3-like isoform X2 | Yes |  |  |  |  |
| - Tropomyosin | Yes | Yes | Lower | No | Low |
| - Tropomyosin | Yes | Yes | Lower | No | Low |
| - Troponin T-like isoform X8 | Yes | Yes | No change | No | Low |
| - Uncharacterised protein LOC111103843 |  | Yes |  |  |  |
| - Uncharacterised protein LOC111121485 | Yes |  |  |  |  |
| - Vitellogenin | Yes | Yes | No change | Some | Low |

**Table S5:** Effective antimicrobial concentrations of cystatins from diverse sources and the isolation methods used to obtain them. Target micro-organisms include human and environmental pathogens.

| **Source** | **Specific cystatin** | **Isolation method** | **Method summary** | **Target species** | **Effective concentrations** | **References** |
| --- | --- | --- | --- | --- | --- | --- |
| **Plant** |  |  |  |  |  |  |
| *Moringa oleifera* | *M. oleifera* phytocystatin (papain inhibitor) | Affinity chromatography | Samples loaded onto a papain-glyoxyl-agarose column connected to an Äkta-Purifier. Affiliated proteins (cystatins) were eluted after complete removal of the non-retained proteins with phosphate buffer. | *Citrobacter amalonaticus, Enterobacter cloacae, Enterococcus faecalis, Escherichia coli, Klebsiella pneumoniae, Proteus vulgaris, Pseudomonas aeruginosa, Salmonella typhimurium*, *Staphylococcus aureus* | 24-175 µg/mL (preliminary) | [23] |
| Sugar cane (*Saccharum officinarum*) | CaneCPI-5 | Recombinant expression in *E. coli*, affinity chromatography, membrane dialysis | The cDNA sequence was obtained by dideoxy sequencing. The coding region was amplified by PCR and inserted into the plasmid pET28 which was used to transform *E. coli*. Cells were stimulated then lysate was collected and centrifuged. Soluble cystatin was purified from the supernatant using an affinity nickel resin column. The column was washed then the protein was eluted with buffer including imidazole. The fractions containing the purified protein were dialysed using MWCO:3 membranes. | *Trichoderma reesei* (filamentous fungi) and mixed-species oral biofilms | 100-200 µg/mL (preliminary) | [24, 25] |
| **Invertebrate** | |  |  |  |  |  |
| Horseshoe crab (*Limulidae* sp.) hemocytes | L-Cystatin (Limulus cystatin) | Size-exclusion chromatography, dialysis, affinity chromatography | Hemocyte lysate was fractionated on a dextran sulfate-Sepharose CL-6B column. The active fraction was dialysed against 50mM sodium citrate then applied to a Cm-papain-Sepharose CL-6B column and eluted with 2M guanidine hydrochloride in buffer. | *E. coli, S. typhimurium,* *Salmonella minnesota*, *K. pneumoniae, S. aureus* | EC_50_ values ranged from 82 to >100 µg/mL | [26] |
| **Vertebrate** |  |  |  |  |  |  |
| Chicken egg white | CEWC (chicken egg white cystatin) | Affinity and size-exclusion chromatography, membrane ultrafiltration | Egg white was purified on S-carboxymethylated papain-Sepharose 4B28. Cystatin-containing fractions were pooled and subject to size exclusion chromatography on Sephadex G-75 then concentrated by ultrafiltration with YM-3 membrane. | *Candida* spp. (including *C. albicans* and *C. alabrata*) and *E. coli* | 0.8 to >26 µM/L | [27] |
| Chicken egg white | Cystatin | Affinity chromatography, dialysis, reverse-phase HPLC | The supernatant was subject to a papain Sepharose 4B column. Bound proteins were eluted with NaOH, the fractions were pooled and concentrated. Concentrate was dialysed against Tris-HCl buffer containing NaCl, and applied to a Sephadex G-100 column. Two fractions were collected and dialysed, then applied to a DEAE-Sephacel column resulting in a single peak. The fraction was concentrated for additional purification by reversed phase HPLC on a Nucleosil 100 C18 column using a Waters HPLC system. The concentrated protein solution was injected onto a C18 (8 100 mm) HPLC column before elution. | *Acinetobacter lwoffii, E. coli, P. aeruginosa, Olgella* sp., *S. enterica Enteritidis, Serratia liquefaciens, Citrobacter freundii,* *S. aureus, Staphylococcus gallinarum*, *Staphylococcus xylosus* | IC_50_ 80-100 µg/mL (preliminary), complete inhibition 100-200 µg/mL | [28] |
| Porcine | CST11 | Recombinant expression in *Pichia pastoris*, membrane dialysis, ion-exchange chromatography | The plasmid was constructed by synthesising porcine CST11 from the mRNA sequence. The product was cloned into the vector pwPICZalpha. The plasmid was transformed into the *P. pastoris* strain X-33. Cells were stimulated, harvested and centrifuged. Supernatant was loaded into a XK 16/20 column packed with Ni-NTA resin. Bound proteins were eluted, fractions containing CST11 were pooled and dialysed using a 3.5-kDa cutoff membrane tubing. The dialysed sample was loaded onto an XK 16/20 column containing strong cation exchange resin. Bound protein was eluted with sodium borate in buffer into eight fractions including rCST11. | *S. aureus, Staphylococcus epidermidis, Bacillus subtilis* | MBCs 17.15–34.27 µM | [29] |
| Human saliva | Cystatin C | Commercially sourced | Human recombinant cystatin C, expressed in *P. pastoris* | *E. faecalis, S. mutans* | 100 µg/mL (preliminary) | [30] |
| Human | Cystatin 9 (rCST9) | Recombinant expression in *Escherichia coli*, dialysis | Primers based on homo sapiens cystatin 9 sequence were used for PCR amplification. PCR products were cloned into the bacterial protein expression plasmid pPROEX. Recombinant plasmids were transformed into *E. coli* BL21 DE3 competent cells, which were then induced. Proteins were eluted using a buffer before being dialysed overnight into 4°C PBS using slide-a-lyser dialysis cassettes with a molecular weight cut-off of 3.5 kDa. | *Francisella tularensis* | 50 pg | [31] |
| Human saliva | Cystatin SA | Affinity and size-exclusion chromatography, dialysis | After clarifying the saliva, it was subjected to an affinity chromatography column with AA as the substrate. The proteins bound to AA were eluted from the column and identified using mass spectrometry (MALDI-TOF/TOF MS). Among other proteins that were bound, cystatin SA was observed in significantly higher concentrations, and this was purified from the eluate via a second size exclusion chromatography step using 15 kDaMWCO filter to select for the 14 kDa cystatin SA. The sample was then dialysed against water. | *Aggregatibacter actinomycetemcomitans* (AA) | 100 µg/mL | [32] |
| Human | Cystatin 9 and Cystatin C | Commercially sourced | Human recombinant cystatins | Multi-drug resistant *K. pneumoniae* | 50-1000 pg | [33] |
| Human | Cystatin C (hCST3) | Transgenic expression in chickens, affinity chromatography | The cystatin C gene codon was ligated onto a piggyBac transposon transgene expression system vector and inserted into chicken primordial germ cells, which were transplanted into recipient chicken embryos. hCST3 expression in transgenic chickens was analysed in egg white and homogenised muscle, and hCST3 was extracted using a His-tagged protein purification kit. Briefly, samples were mixed with Ni-NTA magnetic silica resin and placed on a Nd magnet for 1 min. After washing with binding/washing buffer, elution buffer was added to collect the purified hCST3 eluate. | *E. coli* | 100 ng/disc (preliminary) | [34] |
| Human semen | Cystatin C (Cys C) | Gel-permeation chromatography, dialysis, immunoaffinity chromatography | (i) Fractionation of seminal plasma proteins using a HiLoad 16/60 Superdex-200 column. Eluted fractions were dialysed against water. (ii) Purification of Cys C from fraction-4. Antibodies to commercial Cys C were raised in rabbits. Immunoglobulins (IgG) were purified from rabbit serum were immobilised on the column provided in a kit. The column was washed with the coupling buffer and sodium cyanoborohydride was added. Reactants and uncoupled IgG were washed off. The IgG-coupled column was equilibrated and Fraction-4 (containing Cys C) was added to the column and washed to remove unbound proteins before Cys C bound to the column was eluted. | Human immunodeficiency virus (HIV) | IC_50_ 0.280 μM | [35] |
| Synthetic | Cystapep 1 (Synthetic peptidomimetic structurally based on the N-terminal fragment of human cystatin C) | Chemical synthesis | (2S)-1-amino-2-[N-(tert-butyloxycarbonyl)-amino]-3-methylbutane hydrochloride was prepared from Boc-L-valinol. A series of chemical reactions were performed. The product was isolated using ion-exchange chromatography on a column of S-Sepharose FF and finally purified by reversed phase HPLC. The structure of Cystapep 1 was confirmed by FAB-MS and 1H-NMR. | *S. pyogenes, Streptococcus agalactiae, Streptococcus equisimilis, Streptococcus anginosus, S. pneumoniae, S. aureus, S. epidermis,* clinical isolates of streptococci and staphylococci, *E. faecalis, E. faecium, Listeria monocytogenes, Moraxella catarrhalis, Haemophilus influenzae, E. coli, K. pneumoniae, P. aeruginosa* | 1000 µg/mL preliminary assays; MICs 16 to 256 μg/mL. streptococci most susceptible. Gram neg not susceptible (except *E. coli*) | [36-38] |

**References**

1. Summer, K., J. Browne, L. Liu, and K. Benkendorff. (2020). Molluscan compounds provide drug leads for the treatment and prevention of respiratory disease*.* *Marine Drugs*, *18*(11): 570.

2. Summer, K., L. Liu, Q. Guo, B. Barkla, and K. Benkendorff. (2024). Semi-purified antimicrobial proteins from oyster hemolymph inhibit pneumococcal infection*.* *Marine Biotechnology*. APMBC and ANZMBS joint conference special issue (2024): 1-14.

3. Shen, C., H. Liang, Z. Guo, and M. Zhang. (2023). Members of the histone-derived antimicrobial peptide family from the pearl oyster Pinctada fucata Martensii: Inhibition of bacterial growth*.* *Fish and Shellfish Immunology*, *132*(108439).

4. Notariale, R., A. Basile, E. Montana, N.C. Romano, M.G. Cacciapuoti, F. Aliberti, R. Gesuele, F. De Ruberto, S. Sorbo, G.C. Tenore, M. Guida, K.V. Good, J. Ausio, and M. Piscopo. (2018). Protamine-like proteins have bactericidal activity: The first evidence in Mytilus galloprovincialis*.* *Acta Biochimica Polonica*, *65*(4): 585-594.

5. Domeneghetti, S., M. Franzoi, N. Damiano, R. Norante, N. M. El Halfawy, S. Mammi, O. Marin, M. Bellanda, and P. Venier. (2015). Structural and antimicrobial features of peptides related to myticin c, a special defense molecule from the mediterranean mussel Mytilus galloprovincialis*.* *Journal of Agricultural and Food Chemistry*, *63*(42): 9251-9259.

6. Mitta, G., F. Hubert, T. Noel, and P. Roch. (1999). Myticin, a novel cysteine-rich antimicrobial peptide isolated from haemocytes and plasma of the mussel Mytilus galloprovincialis*.* *European Journal of Biochemistry*, *265*(1): 71-78.

7. Seo, J.K., J.M. Crawford, K.L. Stone, and E.J. Noga. (2005). Purification of a novel arthropod defensin from the American oyster, Crassostrea virginica*.* *Biochemical and Biophysical Research Communications*, *338*(4): 1998-2004.

8. Mao, F., Y. Bao, N.K. Wong, M. Huang, K. Liu, X. Zhang, Z. Yang, W. Yi, X. Shu, Z. Xiang, Z. Yu, and Y. Zhang. (2021). Large-scale plasma peptidomic profiling reveals a novel, nontoxic, Crassostrea hongkongensis-derived antimicrobial peptide against foodborne pathogens*.* *Marine Drugs*, *19*(8).

9. Seo, J.K., M.J. Lee, B.H. Nam, and N.G. Park. (2013). Cgmolluscidin, a novel dibasic residue repeat rich antimicrobial peptide, purified from the gill of the Pacific oyster, Crassostrea gigas*.* *Fish and Shellfish Immunology*, *35*(2): 480-488.

10. Zhang, Y., P. Cui, Y. Wang, and S. Zhang. (2018). Identification and bioactivity analysis of a newly identified defensin from the oyster Magallana gigas*.* *Developmental and Comparative Immunology*, *85*: 177-187.

11. Liu, Z., S. Dong, J. Xu, M. Zeng, H. Song, and Y. Zhao. (2008). Production of cysteine-rich antimicrobial peptide by digestion of oyster (Crassostrea gigas) with alcalase and bromelin*.* *Food Control*, *19*(3): 231-235.

12. Loth, K., A. Vergnes, C. Barreto, S.N. Voisin, H. Meudal, J. Da Silva, A. Bressan, N. Belmadi, E. Bachere, V. Aucagne, C. Cazevielle, H. Marchandin, R.D. Rosa, P. Bulet, L. Touqui, A.F. Delmas, and D. Destoumieux-Garzon. (2019). The ancestral n-terminal domain of big defensins drives bacterially triggered assembly into antimicrobial nanonets*.* *Mbio*, *10*(5).

13. Erdem Büyükkiraz, M. and Z. Kesmen. (2022). Recombinant expression and coexpression of oyster defensin and proline-rich peptide in Komagataella phaffii*.* *Biotechnology and Applied Biochemistry*, *69*(5): 1998-2007.

14. Gueguen, Y., R. Bernard, F. Julie, S. Paulina, D.G. Delphine, V. Franck, B. Philippe, and B. Evelyne. (2009). Oyster hemocytes express a proline-rich peptide displaying synergistic antimicrobial activity with a defensin*.* *Molecular Immunology*, *46*(4): 516-522.

15. Gueguen, Y., A. Herpin, A. Aumelas, J. Garnier, J. Fievet, J.M. Escoubas, P. Bulet, M. Gonzalez, C. Lelong, P. Favrel, and E. Bachère. (2006). Characterization of a defensin from the oyster Crassostrea gigas: Recombinant production, folding, solution structure, antimicrobial activities, and gene expression*.* *Journal of Biological Chemistry*, *281*(1): 313-323.

16. Zhong, J., W. Wang, X. Yang, X. Yan, and R. Liu. (2013). A novel cysteine-rich antimicrobial peptide from the mucus of the snail of Achatina fulica*.* *Peptides*, *39*(1): 1-5.

17. Chand, S. and P. Karuso. (2017). Isolation and total synthesis of two novel metabolites from the fissurellid mollusc Scutus antipodes*.* *Tetrahedron Letters*, *58*(10): 1020-1023.

18. Dolashka, P., A. Dolashki, J. Van Beeumen, M. Floetenmeyer, L. Velkova, S. Stevanovic, and W. Voelter. (2016). Antimicrobial activity of molluscan hemocyanins from Helix and Rapana snails*.* *Current Pharmaceutical Biotechnology*, *17*(3): 263-270.

19. Dolashka, P., V. Moshtanska, V. Borisova, A. Dolashki, S. Stevanovic, T. Dimanov, and W. Voelter. (2011). Antimicrobial proline-rich peptides from the hemolymph of marine snail Rapana venosa*.* *Peptides*, *32*(7): 1477-1483.

20. Gasu, E.N., H.S. Ahor, and L.S. Borquaye. (2019). Peptide mix from Olivancillaria hiatula interferes with cell-to-cell communication in pseudomonas aeruginosa*.* *BioMed Research International*, *2019*.

21. Bitaab, M.A., S.O. Ranaei Siadat, J. Pazooki, and Y. Sefidbakht. (2015). Antibacterial and molecular dynamics study of the dolabellanin b2 isolated from sea slug, Peronia peronii*.* *Biosciences Biotechnology Research Asia*, *12*(3): 2023-2035.

22. Miller, B.W., J.P. Torres, J.O. Tun, M.S. Flores, I. Forteza, G. Rosenberg, M.G. Haygood, E.W. Schmidt, and G.P. Concepcion. (2020). Synergistic anti-methicillin-resistant Staphylococcus aureus (MRSA) activity and absolute stereochemistry of 7,8-dideoxygriseorhodin c*.* *Journal of Antibiotics*, *73*: 290-298.

23. Cotabarren, J., S. Claver, J.A. Payrol, J. Garcia-Pardo, and W.D. Obregón. (2021). Purification and characterization of a novel thermostable papain inhibitor from Moringa oleifera with antimicrobial and anticoagulant properties*.* *Pharmaceutics*, *13*(4).

24. Soares-Costa, A., L.M. Beltramini, O.H. Thiemann, and F. Henrique-Silva. (2002). A sugarcane cystatin: Recombinant expression, purification, and antifungal activity*.* *Biochemical and Biophysical Research Communications*, *296*(5): 1194-1199.

25. Pelá, V.T., A.S. Braga, G.D. Camiloti, J.G.Q. Lunardelli, J.G. Pires, D. Toyama, A.C. Santiago, F. Henrique-Silva, A.C. Magalhães, and M.A.R. Buzalaf. (2021). Antimicrobial and anti-caries effects of a novel cystatin from sugarcane on saliva-derived multi-species biofilms*.* *Swiss dental journal*, *131*(5): 410-416.

26. Agarwala, K.L., S. Kawabata, M. Hirata, M. Miyagi, S. Tsunasawa, and S. Iwanaga. (1996). A cysteine protease inhibitor stored in the large granules of horseshoe crab hemocytes: Purification, characterization, cDNA cloning and tissue localization*.* *Journal of Biochemistry*, *119*(1): 85-94.

27. Kolaczkowska, A., M. Kolaczkowski, A. Sokolowska, H. Miecznikowska, A. Kubiak, K. Rolka, and A. Polanowski. (2010). The antifungal properties of chicken egg cystatin against candida yeast isolates showing different levels of azole resistance*.* *Mycoses*, *53*(4): 314-320.

28. Wesierska, E., Y. Saleh, T. Trziszka, W. Kopec, M. Siewinski, and K. Korzekwa. (2005). Antimicrobial activity of chicken egg white cystatin*.* *World Journal of Microbiology and Biotechnology*, *21*(1): 59-64.

29. Fan, K., J. Jiang, Z. Wang, R. Fan, W. Yin, Y. Sun, and H. Li. (2014). Expression and purification of soluble porcine cystatin 11 in pichia pastoris*.* *Applied Biochemistry and Biotechnology*, *174*(5): 1959-1968.

30. Blancas, B., M.D.L. Lanzagorta, L.F. Jiménez-Garcia, R. Lara, J.L. Molinari, and A.M. Fernández. (2021). Study of the ultrastructure of Enterococcus faecalis and Streptococcus mutans incubated with salivary antimicrobial peptides*.* *Clinical and Experimental Dental Research*, *7*(3): 365-375.

31. Eaves-Pyles, T., J. Patel, E. Arigi, Y. Cong, A. Cao, N. Garg, M. Dhiman, R.B. Pyles, B. Arulanandam, A.L. Miller, V.L. Popov, L. Soong, E.D. Carlsen, C. Coletta, C. Szabo, and I.C. Almeida. (2013). Immunomodulatory and antibacterial effects of cystatin 9 against Francisella tularensis*.* *Molecular Medicine*, *19*(1): 263-275.

32. Ganeshnarayan, K., K. Velliyagounder, D. Furgang, and D.H. Fine. (2012). Human salivary cystatin SA exhibits antimicrobial effect against Aggregatibacter actinomycetemcomitans*.* *Journal of Periodontal Research*, *47*(5): 661-673.

33. Holloway, A.J., J. Yu, B.P. Arulanandam, S.M. Hoskinson, and T. Eaves-Pyles. (2018). Cystatins 9 and c as a novel immunotherapy treatment that protects against multidrug-resistant New Delhi metallo-beta-lactamase-1-producing Klebsiella pneumoniae*.* *Antimicrobial Agents and Chemotherapy*, *62*(3).

34. Kim, S.W., J.H. Lee, J.S. Han, S.P. Shin, and T.S. Park. (2021). Piggybac transposition and the expression of human cystatin c in transgenic chickens*.* *Animals*, *11*(6).

35. Vernekar, V., S. Velhal, and A. Bandivdekar. (2015). Evaluation of cystatin c activities against HIV*.* *Indian Journal of Medical Research*, *142*(April): 423-430.

36. Jasir, A., F. Kasprzykowski, R. Kasprzykowska, V. Lindström, C. Schalen, and A. Grubb. (2003). New antimicrobial cystatin c-based peptide active against gram-positive bacterial pathogens, including methicillin-resistant Staphylococcus aureus and multiresistant coagulase-negative staphylococci*.* *APMIS*, *111*(11): 1004-1010.

37. Jasir, A., F. Kasprzykowski, V. Lindström, C. Schalén, and A. Grubb. (2004). New antimicrobial peptide active against gram-positive pathogens*.* *Indian Journal of Medical Research, Supplement*, *119*(May): 74-76.

38. Pikuła, M., M. Smużyńska, A. Krzystyniak, M. Zieliński, P. Langa, M. Deptuła, A. Schumacher, J. Łata, M. Cichorek, A. Grubb, P. Trzonkowski, F. Kasprzykowski, and S. Rodziewicz-Motowidło. (2017). Cystatin c peptidomimetic derivative with antimicrobial properties as a potential compound against wound infections*.* *Bioorganic and Medicinal Chemistry*, *25*(4): 1431-1439.
