# Supplementary material for: Antimicrobial proteins from oyster hemolymph improve the efficacy of conventional antibiotics
Source: PLoS One. 2025 Jan 21;20(1):e0312305. doi: 10.1371/journal.pone.0312305 (PMC11750097; doi:10.1371/journal.pone.0312305)
Supplement: S2 File — (DOCX) [file pone.0312305.s002.docx]

**Antimicrobial proteins from oyster hemolymph improve the efficacy of conventional antibiotics**

Kate Summer, Lei Liu, Qi Guo, Sarah Giles, Bronwyn Barkla, and Kirsten Benkendorff

**SUPPLEMENTARY FIGURES**

**
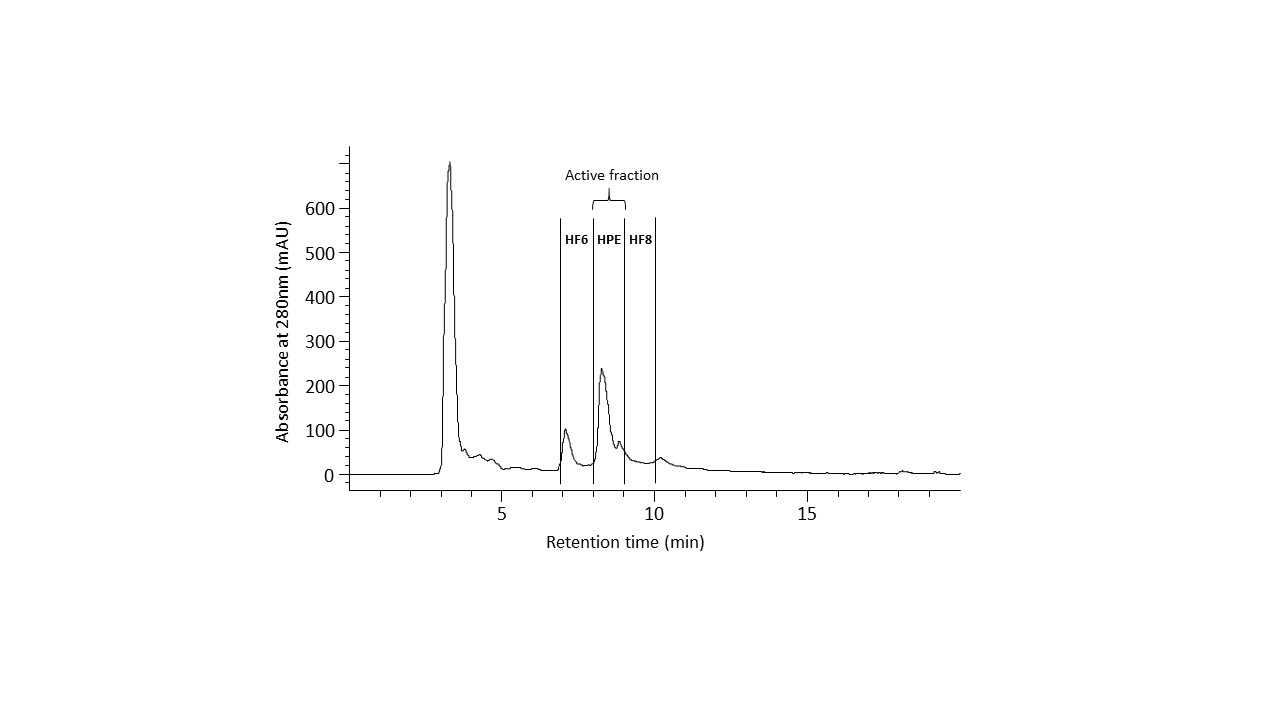
**

**Fig S1:** Analysis of a typical SRO cell-free hemolymph sample obtained by preparative HPLC. A) The chromatogram at 280 nm shows the relative composition of hemolymph components across the three fractions collected between 7-10 mins at 1 min time slices, where HPE showed strong antibacterial-antibiofilm activity and HF6 and HF8 were relatively inactive. B) Full UV spectra of the active fraction. C) UV spectra of the active fraction at relevant wavelengths. Absorbance at 280 nm is specific to proteins.

mAU

Wavelength (nm)

**B**

**C**

278 nm

**A**


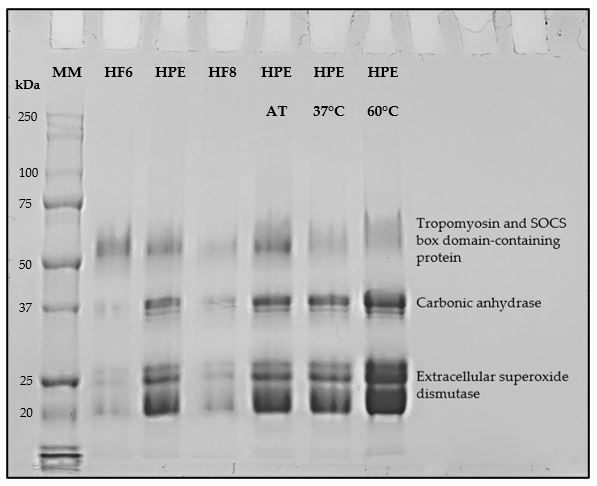


**Fig S2:** SDS-PAGE runs of HPLC-fractionated SRO cell-free hemolymph. HFR1-3 were stored at -80°C until testing. HPE was tested for stability after storage at ambient laboratory temperature (AT) for 24 h, 37°C for 1 h, and 60°C for 1 h. Samples were prepared to 10 µL in Laemmli buffer representing 1-2 g total protein. Molecular marker (MM) was positioned in the first well of each gel and buffer was added to empty wells. Proteins in each band were identified by HPLC-MS/MS .

**
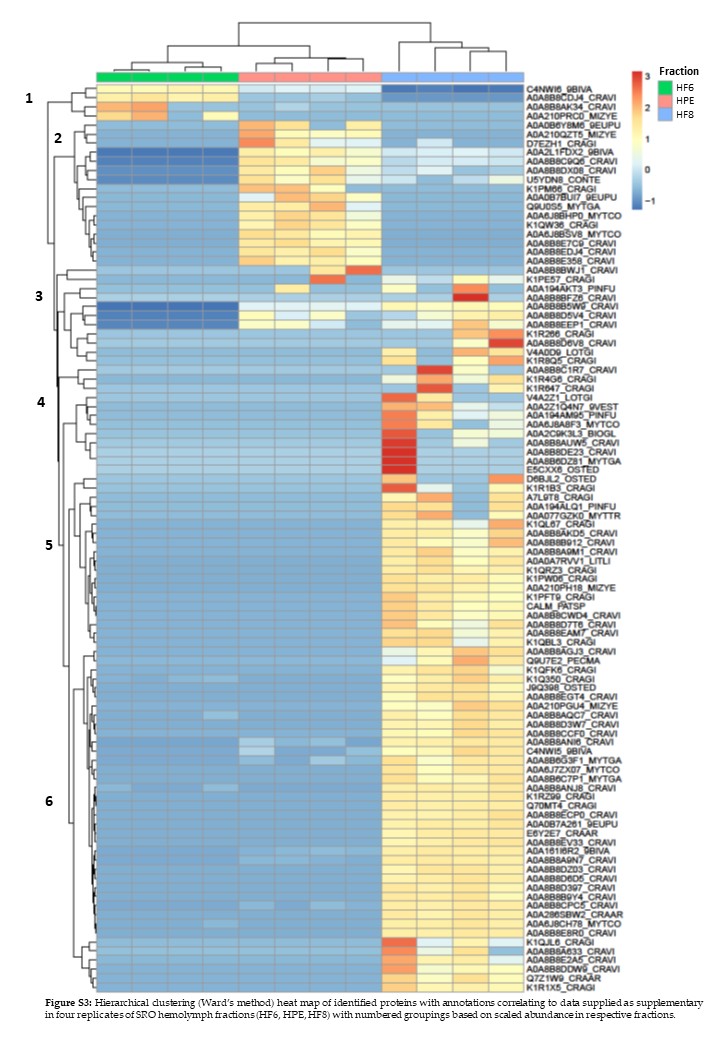
**

**Fig S3:** Hierarchical clustering (Ward’s method) heat map of identified proteins with annotations correlating to data supplied as supplementary in four replicates of SRO hemolymph fractions (HF6, HPE, HF8) with numbered groupings based on scaled abundance in respective fractions.

**Fig S4:** Hierarchical clustering (Ward’s method) heat map of identified proteins with annotations correlating to data supplied as supplementary in four replicates of SRO hemolymph fractions (HF6, HPE, HF8) with numbered groupings based on scaled abundance in respective fractions.

**
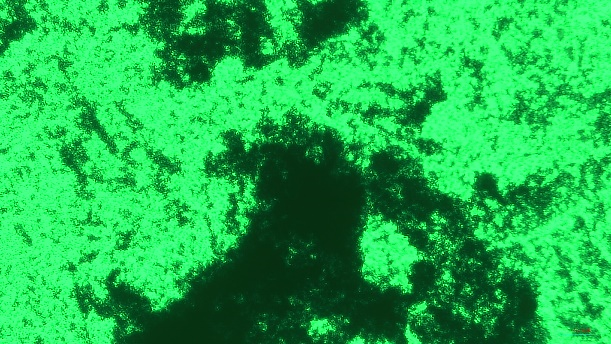

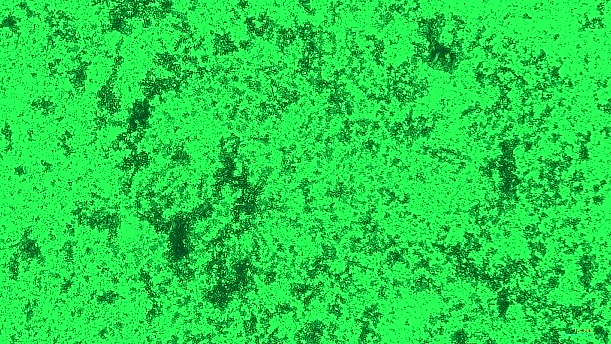

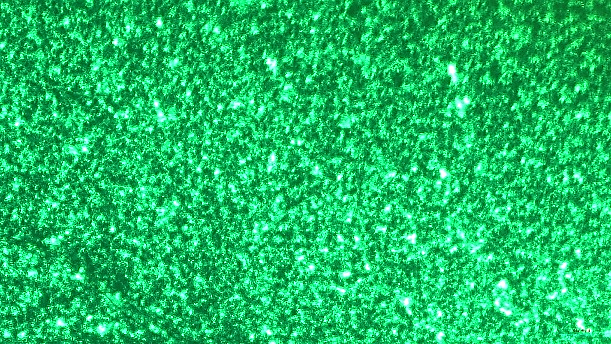

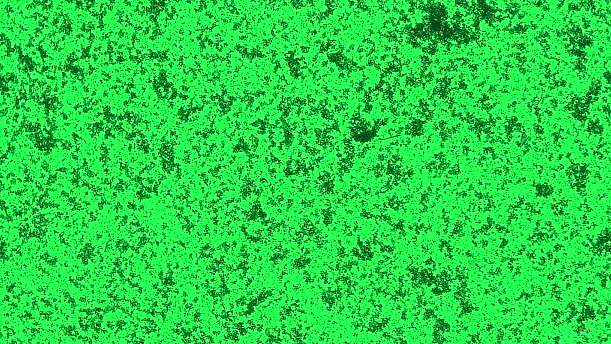

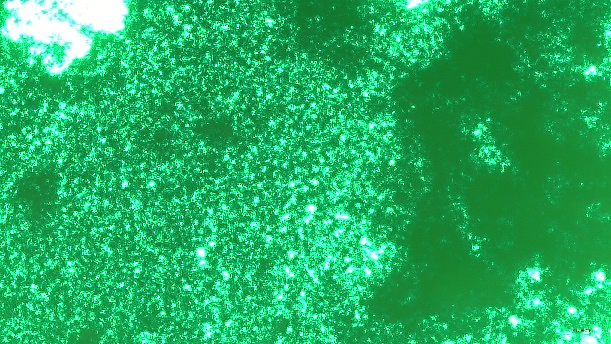

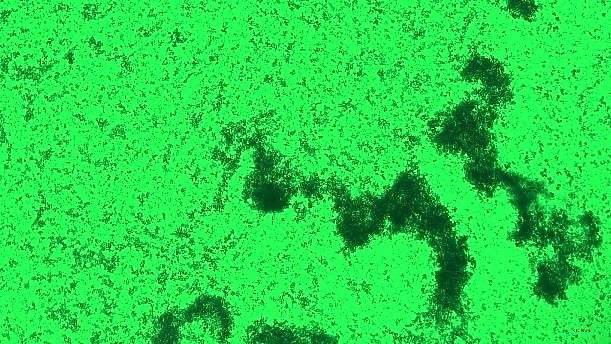

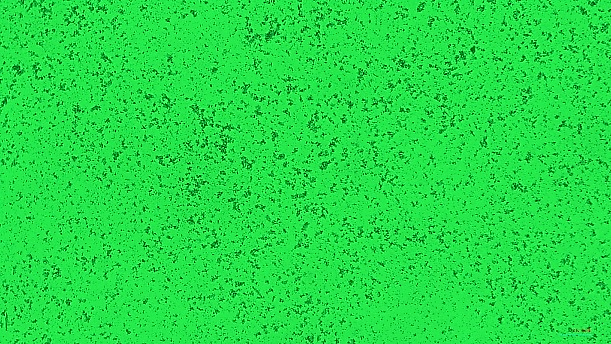

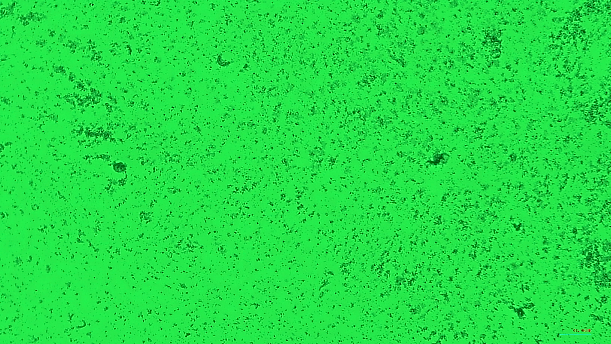

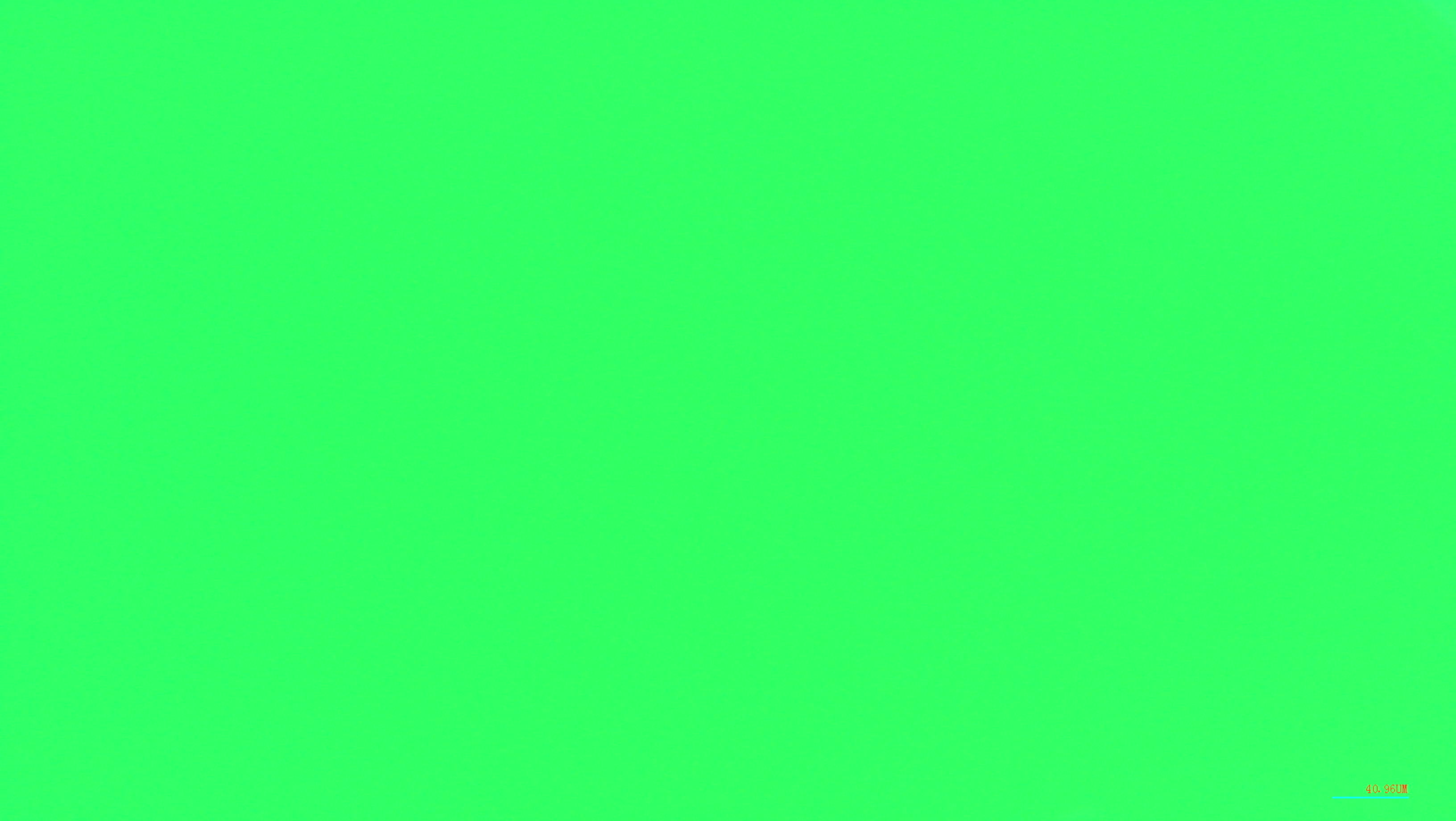
**

**A**

**B**

**C**

ATCC 51916

Clinical - T19F

**D**

Clinical - SKB

Clinical – T6B

**1 (100% growth)**

**2 (50% growth)**

**E**

MBC for all species and strains

100 µm

100 µm

100 µm

100 µm

100 µm

100 µm

100 µm

**Fig S5:** Light microscopic images (at 100×) of *Streptococcus pneumoniae* biofilm formations vary between strains (A-D), but all are susceptible to HPE. Plates in column 1 are positive growth controls (no treatment), plates in column 2 are treated with an intermediate concentration of HPE (reduced growth). Wells at MBC concentrations of HPE show no growth for all strains (E). Biofilms were stained with crystal violet before images were taken under green light at 20× magnification using a Nikon Eclipse Ts2 inverted microscope.
