# Supplementary material for: Antimicrobial proteins from oyster hemolymph improve the efficacy of conventional antibiotics
Source: PLoS One. 2025 Jan 21;20(1):e0312305. doi: 10.1371/journal.pone.0312305 (PMC11750097; doi:10.1371/journal.pone.0312305)
Supplement: Attachment — Submitted filename: Author responses to reviewer comments_final.docx [file pone.0312305.s004.docx]

| **Antimicrobial proteins from oyster hemolymph improve the efficacy of conventional antibiotics**  Kate Summer, Qi Guo, Lei Liu, Bronwyn Barkla, Sarah Giles, Kirsten Benkendorff  **Author responses to reviewer comments** | |
| --- | --- |
| **Reviewer comments** | **Author responses** |
| **REVIEWER 1** |  |
| 2.1 HPE Preparation Hemolymph Collection from SRO (Section 2.1.1): Ensure that the referenced method [36] is accessible and sufficiently detailed for replication. The statement "Hemolymph was withdrawn from multiple oysters, combined to obtain 5 mL pools" would benefit from specifying how many oysters were used on average per pool, as this could impact the consistency and yield of hemolymph. | Wording has been changed to clarify (Lines 74-76). The level of detail should allow other researchers to repeat the procedure and the references indicate that these are the same procedures as used in previous studies. Reference 36 has now also been updated.  The average number of oysters per pool is now specified (line 77).  (please note, line numbers are provided for the document with tracked changes on) |
| Hemolymph Fractionation (Section 2.1.2):  The freeze-drying process is well-explained, but adding details about the reconstitution process (e.g., the volume of Milli-Q water used) would improve clarity. | The reconstitution volume (500µl)_ is now provided (line 94). Phosphate buffered saline was used to reconstitute the protein fractions. |
| HPE Heat Treatments (Section 2.1.3):  The heat treatment conditions are well-documented. It would be beneficial to include a brief rationale for the chosen temperatures and durations to understand their relevance to the study's objectives. | A brief rationale for the chosen temperatures is now provided.  “These temperatures and exposure durations were selected to represent conditions that may be encountered during experimentation or handling e.g., thawed/laboratory temperature, body temperature, overheating during transport” (lines 100-102) |
| 2.2 Proteomics Protein Quantification and Visualization (Section 2.2.1): Ensure that the sources of all reagents and materials are consistently specified (e.g., "Bio-Rad, Australia" for the protein dye reagent). | Sources of PBS and BSA are now specified (lines 108-109). |
| Protein Identification by HPLC-MS/MS (Section 2.2.2): Mentioning the specific version of the database used for searching spectra (e.g., the release date or version number of the Uniprot Mollusca database) would enhance reproducibility. The protein extraction and digestion process is well-described, but clarifying the final volume in which peptides are redissolved before analysis could prevent confusion. | The number of entries in 2024 and a reference for the UniProt database is now provided [33] (line 151).  The final volume of 15µL is supplied in line 133. |
| Protein Data Analysis (Section 2.2.3): Including an explanation for choosing NSAF and the specific clustering methods (PCA and hierarchical clustering) would provide context for readers unfamiliar with these techniques. | Explanations for choosing NSAF quantitative analysis and the clustering methods are now provided, with references as follows:  “NSAF normalises spectral counts by protein length, ensuring accurate relative abundance measurements. This method is simple and robust, and its log-transformed values facilitate statistical analyses, allowing for effective cross-sample comparisons”(lines 153-156).  “These clustering methods are generally applied to analyse and visualise the relationships and differences between samples based on the quantified data.” (Lines 158-159) |
| 2.3 Antibacterial and Antibiofilm Assays Media and Reagents (Section 2.3.1): Ensure that any deviations from standard protocols (if any) are mentioned. | Standard protocols were used for the preparation of media and reagents. There are no deviations from standard protocols in the preparation of media or reagents. |
| Bacteria Preparation (Section 2.3.2): The preparation of bacterial cultures is detailed and aligns with standard procedures. Specifying the rationale for selecting the particular strains and clinical isolates would add context. | The rationale for selecting the specific strains and isolates is because they are highly representative of common clinical infections, which is now stated in lines 179-180 after justification for selection of the bacterial species in lines 171-179. Additional details for the relevance of each strain have been added to Supplementary Table S1. |
| Antibacterial-biofilm Inhibition Coupled Assays (Section 2.3.3): The assay setup and conditions are well-documented. The choice of concentrations for HPE and antibiotics could be briefly justified to highlight their relevance to the study. The statistical methods used for data analysis are appropriate. Consider providing additional details on the criteria for determining significant differences. | “The treatment concentration range was chosen to incorporate effective concentrations expected on the basis of previous work [30].” Is now stated (lines 224-226).  Additional details on criteria for determining significance from dose-response models is now provided (lines 270-271):  “Significance (p<0.05) was assessed based on whether zero was contained within the 95% credible intervals (CI’s).” |
| Biofilm Treatment Assays (Section 2.3.4): It would be helpful to explain why specific controls were chosen (e.g., EDTA and ciprofloxacin) to contextualize their relevance as benchmarks. | We now indicate that EDTA and ciprofloxacin are known antibiofilm agents, with references. (line 229) |
| 2.4 Cytotoxicity Assays Cell Viability Using the MTS Assay (Section 2.4.3): Justifying the range of HPE concentrations tested would add value, as would a brief discussion on the selection of statistical methods for data analysis. | We have now included a brief justification for the range of HPE concentrations used based on effective concentrations in the antimicrobial assays (lines 267-268).  ANOVA is a standard approach for comparing means between different treatment groups. It is considered a robust approach for analysis of cytotoxicity data and our data met the assumptions of normality and equal distributions for parametric analysis —this is now stated in the text supported by two references (lines 275-277). |
| Antibacterial Efficacy: Provide detailed comparisons with other AMPs. | We originally made comparisons between HPE and approved AMPs in lines 488-494; HPE (containing cystatins) is also compared to cystatins from other sources in paragraph beginning line 509. Lines 492-499 now expand on comparisons to other AMPs. Detailed comparisons between HPE and other molluscan natural products/AMPS are now provided with the addition of Supplementary Table S4 (referred to in line 499). |
| Synergistic Effects: Expand on mechanisms and cite relevant studies. | In lines 456-460, we propose mechanisms of action for combination effects and cite relevant studies, including interference with cell membrane integrity allowing antibiotics to access the cells, as well as potential independent effects like direct enzyme inhibition. We also discuss the mechanisms of action of cystatins (509-529) and carbonic anhydrase (530-533). Since mechanisms of action were not tested in this study, any further discussion would be highly speculative. |
| Clinical Relevance: Discuss potential integration into clinical protocols. | Clinical integration is a consideration more relevant to later stages of the drug development pipeline. We think that discussing potential integration into clinical protocols would be too speculative and thus not helpful at this stage. We have however summarised the next stages required for therapeutic development (see new Figure 10) |
| Challenges: Elaborate on production costs and efficacy barriers. | Thank you for this suggestion. We have elaborated on production costs, opportunities, barriers and processes throughout lines 556-594. This includes “strict regulations and requirements for clinical testing, quality control and registration” as well as opportunities for sustainable supply through aquaculture production and investment in technology for value-adding via high throughput extraction. |
| Future Research: Specify next steps and potential collaborative efforts. | We have now specified next steps and opportunities for pharmaceutical/research and aquaculture industries in the new paragraphs mentioned (line numbers as above) and in Figure 10. Further development will require collaboration between researchers, regulators and industry, including the oyster aquaculture for production and the marine biotech industry for value-adding and quality control. |
| **REVIEWER 2** |  |
| I highly recommended the manuscript and have a future scope. Some grammar and spelling mistakes should be removed in revised thesis. | Thank you for bringing our attention to spelling and grammar errors. Spelling and grammar checks have now been completed with minor changes throughout. |
| **REVIEWER 3** |  |
| 1. Line 154: Correct the sign “°C”. | Degrees sign has been corrected |
| 2. Line 155: correct “up to 30 50ms” | Corrected by removing “up to 30” (this was unnecessary) |
| 3. Figure 1 should be improved. The authors can use simply PPT to make the labelling in the figure. | Thank you for this suggestion. Labels were generated using PPT and the Figure is now provided in high resolution. |
